# High risk men who have sex with men in Spain are reporting low intentions of actively seeking HIV testing: results from a cross-sectional study

**DOI:** 10.1186/s12889-020-8440-3

**Published:** 2020-03-27

**Authors:** Juan Hoyos, Kostas Koutentakis, Tomás Maté, Jose Pulido, Luis Sordo, Juan-Miguel Guerras, María-José Belza

**Affiliations:** 1 Consortium for Biomedical Research in Epidemiology & Public Health (CIBERESP), Madrid, Spain; 2 Department of Public Health and Maternal and Child Health, Madrid Complutense University, Madrid, Spain; 3 Carlos III Health Institute, National Center of epidemiology, Madrid, Spain; 4 Primary Health Care Management of East Valladolid, Valladolid, Spain; 5 Department of Health Programs, Carlos III Health Institute, National School of Health, Madrid, Spain; 6 Department of Biostatistics and Epidemiology, Carlos III Health Institute, National School of Health, Madrid, Spain

**Keywords:** HIV, Diagnosis, Sexual behaviours

## Abstract

**Background:**

We analyse unprotected anal intercourses (UAI) self-reported by a sample of men who have sex with men (MSM), by their future testing intentions and past testing history to identify undertested subpopulations that could be contributing to onward transmission.

**Methods:**

We recruited MSM through gay dating websites in Spain from September 2012 to April 2013. For MSM at risk of acquiring or unknowingly transmitting HIV (at risk hereafter) we calculate time at risk, number of UAI in the last 12 months and last 5 years by testing intention (low intention (LI), Medium intention (MI), high intention (HI)) and past testing history. For never testers we analyse the reasons for not having been tested.

**Results:**

Of 3272 MSM at risk, 19.8% reported LI of testing. MSM with LI reported the longest period at risk (8.49 years (*p* < 0.001)) and reported 3.20 UAI/person in the last 12 months (vs. 3.23 and 2.56 in MSM with HI and MI (*p* < 0.001)) and 12.90 UAI/person in the last 5 years (vs. 8.07 and 9.82 in MSM with HI and MI (*p* < 0.001)). Those with LI accounted for 21 and 27% of all the UA acts occurring in the last 12 months and the last 5 years. Among never testers (40.6%), those with LI reported lower risk perception (*p* = 0.006).

**Conclusion:**

We identified a group of high risk and undertested MSM that could be behind a substantial proportion of the UAIs with potential of transmission/acquisition of HIV. Given their low willingness to seek an HIV test and low risk perception, they constitute a population that will probably require approaches other than client initiated strategies.

## Background

In Spain, the number of diagnosed HIV infections in men who have sex with men (MSM) has stabilised in recent years [1] . In 2015 38.4% of the newly diagnosed MSM had a CD4 count of <350mm^3^. Furthermore, studies conducted in countries with similar epidemics show that a non-negligible fraction of the MSM population remains undiagnosed [2, 3].

Uncovering the undiagnosed fraction of the epidemic is important because treatment reduces morbidity and mortality [4, 5] and transmission from one individual to another [6, 7]. Currently, the World Health Organisation guidelines recommend that treatment should be initiated when CD4 counts fall to <=500 cells/*mm*^*3*^ or immediately to individuals in sero-discordant relationships [8]. Hence, it is not surprising that the promotion of HIV testing in MSM has been one of the cornerstones of preventive strategies [9].

However, testing rates in MSM are far from fulfilling the current European Centre for Disease Prevention and Control (ECDC) recommendation of testing at least once every 12 months [9] and studies describe a substantial percentage of MSM that have never even been tested for HIV [10–15]. Historically, the primary model for providing HIV testing has been client-initiated HIV testing (CIT) in which individuals must actively seek an HIV test at a health or community-based facility.

Social cognition models, such as the theory of the planned behaviour [16] consider that the likelihood of carrying out a behaviour depends on the strength of the intention. Applied to HIV testing, this means that CIT is linked to the strength of testing intentions as evidence indeed suggests [17]. If an individual has low intentions of actively seeking an HIV test, these strategies might not be effective [18].

In spite of its importance, intention to seek an HIV test among undertested MSM has rarely been studied in developed countries. Most of the studies in this context have been carried out in the US [19] and very few have been conducted in Europe [20, 21] . We have very little information on the size of this subpopulation and on the role they could be playing in onward transmission. This is crucial as it would help policymakers to establish priorities and would provide them with valuable data to design tailored HIV testing strategies.

With this in mind, we aim to identify individuals at risk of acquiring or unknowingly transmitting (AUT) HIV that do not meet current testing recommendations to analyse them by testing intentions and by past testing history. Additionally, we will also analyse their main characteristics and the reasons for not testing for HIV among those who had never been tested before.

## Methods

### Type of study

Online based cross-sectional study.

### Recruitment

Study participants were recruited from September 2012 to April 2013 through recruitment banners in Spanish language allocated mainly in gay dating websites. These websites, connect users based on their physical proximity. Similarly, they are able to limit the visualisation of banners based on geographical localisation. Location based advertising has been utilised for over a decade and uses a number of methods to determine the physical location of the targeted population, namely: Internet protocol address (IP) and GPS. We asked the gay dating websites, to show the promotion banner only to users accessing from Spain. Those who clicked on it, were routed to a secured website.

Potential participants were asked to read an informed consent explaining that the questionnaire was anonymous and confidential and that it took approximately 15 min to complete. In order to proceed, they were asked to click an “I agree” button. Electronic identifiable information and IP were not collected.

### Data collection instrument

We developed an online web-based online questionnaire in Spanish that included sections to assess socio-demographic characteristics, sexual orientation, relation with gay culture, number of unprotected anal intercourses (UAI) in the last 12 months, HIV status, HIV testing history and future testing intentions. Testing intention was assessed with the question: "How likely are you to seek an HIV test in the following 12 months? Based on it, we created a three category variable: very likely/likely (high intention); Not sure (medium intention) and unlikely/highly unlikely to test (low intention). To access the questionnaire content see Additional file 1.

### Inclusion criteria

We required participants to be male at birth, > = 18 years of age and have had sex with another man at least once. We only included MSM who self-reported being HIV negative and those who had never been tested.

### Data analysis

Of 8380 participants meeting the inclusion criteria, we focused our analysis on the *N* = 4255 MSM of whom we could ascertain whether they were at risk of having AUT HIV at the moment of completing the survey. This classification was carried out by using the variables “UAI in the last 12 months” and testing history. We considered MSM at risk of AUT HIV as those who:

- Received their last negative HIV test more than 12 months ago and reported one or more UAI in the last 12 months.
- Had never been tested and reported UAI in the last 12 months.

Likewise, we considered MSM not being at risk AUT HIV as men who:

- Received the last negative test in the last 12 months and reported no UAI in the last 12 months.

*N* = 4125 individuals were not included in the analysis because we were not able to determine their risk of AUT HIV at the moment of the survey:

- *N* = 1738 reported both testing negative and having ≥1 UAI in the last 12 months. In these individuals, we could not assess which came first: UAI or testing and thus we could not assess if they were at risk.
- *N* = 2387 never testers or tested > 12 months ago with no UAIs in the last 12 months were also considered unclassifiable.

A description of these individuals can be found in Additional file 2.

We first carried out a descriptive analysis by risk of AUT HIV and examined differences using chi-square tests.

A second level of analysis, focused exclusively on those individuals at risk of AUT HIV. For this group we first perform a descriptive analysis by testing intention. Differences were assessed using the Chi-square test.

We estimated **mean time at AUT HIV** defined as: time since last HIV negative test (in those who had received a test in the past) or time since first sex with another man in those with no previous HIV tests.

We also estimated the **mean number of at risk UAI per person in the last 12 months** and **at risk UAI per person in the last 5 years**. Number of UAI in the last 5 years was not asked in the questionnaire. For this estimation, we assumed consistent behaviour and extrapolated behaviour in the last 12 months to the remaining 4 years with no information. Calculation depended on testing history and time since first sex with another men:

- Never testers whose first sex with another man was > 5 years ago: at risk UAI per person in the last 12 months * 5 (years)
- Never testers whose first sex with another man was < 5 years ago: at risk UAI per person in the last 12 months * mean time at risk of AUT (years since first sex with another man)
- Participants tested > 5 years ago whose first sex with another man was > 5 years ago: at risk UAI per person in the last 12 months * mean time at risk of AUT (years since first sex with another man)
- Participants tested > 5 years ago whose first sex with another was < 5 years ago: at risk UAI per person in the last 12 months * 5 (years)
- Tested < 5 years ago: at risk UAI per person in the last 12 months * mean time at risk of AUT (time since last test given that first sex with another man was always previous to the last test)

All three estimations (mean time at risk of AUT HIV, mean UAIs per person in the last 12 months and in the last 5 years) were analysed by testing intentions and testing history. The differences were analysed using Kruskal-Wallis non parametric tests for non-normal distributions.

Finally, we calculated the **total number of at risk UAI acts in the last 12 months a**nd **the total number of at risk UAI acts in the last 5 years** as the summation (*Σ)* of all the at risk UAI occurred in the last 12 months reported by the participants. These results were stratified by testing intention.

For MSM who had never been tested before, we investigated the main reason for never testing through a multiple-choice question with 11 closed possible answers and one open-ended answer. We stratified data by testing intention and assessed differences using the Chi-square test.

## Results

### Main characteristics by risk of AUT HIV

Of the 4255 participants of whom we could ascertain risk of AUT HIV, 77.8% (*n* = 3311) where at risk. Briefly, men at risk were younger, less educated (51.3% < university education) and lived in smaller municipalities than those not at risk. At risk individuals were also less involved with the gay scene. The proportion of MSM at risk reporting having low (19.8%) or medium (29.2%) intentions of actively seeking an HIV test in the next 12 months was higher than in the “not at risk” group (see Additional file 2).

### Characteristics of participants at risk of AUT HIV by testing intention

Participants at risk of AUT HIV reporting low testing intentions were younger (49.8% < 30 years of age (*p* < 0.001)), had a higher presence of Spanish born individuals (94.1%) (*p* < 0.001) and reported not being related to the gay scene (56.1%) more frequently than the other two groups (*p* < 0.001) (Table 1).

**Table 1:** Main characteristics of participants at risk of acquiring or transmitting HIV by testing intentions

	High intention (***N*** = 1681)		Medium intention (***N*** = 969)		Low intention (***N*** = 657)		*p*
	N	%	%	%	N	%	
**Age**							< 0.001
< 25	311	18,5	224	23,1	193	29,4	
25–29	321	19,1	204	21,1	134	20,4	
30–39	563	33,5	287	29,6	168	25,6	
40–44	206	12,3	92	9,5	75	11,4	
> =45	280	16,7	162	16,7	87	13,2	
**Place of birth**							< 0.001
Spain	1401	83,3	868	89,6	618	94,1	
Latin-America	174	10,4	63	6,5	22	3,3	
Other Country	106	6,3	38	3,9	17	2,6	
**Study level**							< 0.001
< University	820	49,0	561	58,2	309	47,1	
> University	854	51,0	403	41,8	347	52,9	
**Relationship with gay culture**							< 0.001
Related to the gay scene	1100	66,5	472	50,3	282	43,9	
Not related to gay scene	554	33,5	466	49,7	361	56,1	
**Sexual orientation**							< 0.001
Homosexual	1457	86,7	775	80,4	544	83,1	
Hetero-bisexual	223	13,3	189	19,6	111	16,9	
**Inhabitants in place of residence**							< 0.001
> 1.000.000	474	28,6	202	21,0	132	20,4	
> 500.000–1.000.000	182	11,0	99	10,3	95	14,7	
> 10.000–500.000	841	50,7	545	56,7	333	51,5	
< 10.000	162	9,8	115	12,0	86	13,3	

*pvalue: Chi-square test

### Testing history and time at risk of AUT HIV

The proportion of individuals who reported never having received an HIV test before or who received their last one > 5 years ago was higher in the low intention group (59.6 and 9.9% respectively), than in the medium (54.0 and 8.3%) and high intention groups (24.5 and 4.9%) (*p* < 0.001) (Table 2).

**Table 2:** Time at risk of acquiring/transmitting HIV, number of at risk unprotected anal intercourses per person (UAI) in the last 12 months and 5 years, among MSM at risk of acquiring transmitting HIV

	(***N*** = 3272)a	%	Mean time at risk (years) of acquiring/unadvertidly tansmitting HIV	Mean UAIs per person (last 12 months)	Mean UAIs per person (last 5 years)
**TOTAL**			6,36	3,02	9,54
**TESTING INTENTIONS**	(N = 3272)a		(*p* < 0.001)	(*p* < 0.001)	(*p* < 0.001)
**HIGH**	1670	51,0	4,49	3,23	8,07
**MEDIUM**	954	29,2	8,21	2,56	9,82
**LOW**	648	19,8	8,49	3,20	12,90
**HIGH TESTING INTENTIONS**	(*N* = 1670)				
**Years since last test**			(< 0.001)	(*p* = 0.548)	(< 0.001)
> =1 to < 2	638	38,2	1,08	3,57	3,90
2 to < 5	541	32,4	2,66	3,04	7,89
5 or more	82	4,9	10,05	3,70	18,48
Never tested	409	24,5	11,11	2,85	12,74
**MEDIUM TESTING INTENTIONS**	(*N* = 954)				
**Years since last test**			(< 0.001)	(*p* = 0.179)	(< 0.001)
> =1 to < 2	117	12,3	1,10	2,29	2,50
2 to < 5	243	25,5	3,12	2,69	8,15
5 or more	79	8,3	9,23	2,92	14,62
Never tested	515	54,0	12,07	2,50	11,54
**LOW TESTING INTENTIONS**	(*N* = 648)				
**Years since last test**			(*p*< 0.001)	(*p*=0.004)	(*p*< 0.001)
> =1 to < 2	57	8,8	1,06	3,99	4,06
2 to < 5	141	21,8	3,25	1,99	6,46
5 or more	64	9,9	10,47	4,48	22,38
Never tested	386	59,6	11,17	3,31	14,99

a35 individuals are not included in this analysis because they had missing data on past testing history or number of UAIs

The overall time at risk of AUT was 6.36 years. When analysed by testing intentions, it was higher in those with low (8.49 years) and medium intentions (8.21) than in those with high (4.49) (*p* < 0.001) (Table 2).

### At risk UAI per person: last 12 months and last 5 years

Overall, at risk men reported an average of 3.02 at risk UAI in the last 12 months. Analysed by testing intentions, the number was higher among those who reported high and low intentions of testing (3.23 and 3.20 UAI per person respectively) than in those with medium testing intentions (*p* < 0.001) (Table 2). In the low testing intention group, we found statistically significant differences by testing history (*p* = 0.004) (Table 2).

When we expanded the analysis to the last 5 years, we observed an overall at risk UAI per person of 9.54. By testing intentions, it was higher in the low intention group (12.9 at risk UAI per person) than in the medium (9.82) and high intention groups (8.07) (*p* < 0.001). The number of UAI was higher among those tested > 5 years ago and in never testers. This was consistent across all the three testing intention groups (*p* < 0.001) (Table 2).

### Total UAI acts at risk of acquisition and transmission

**Fig. 1:**
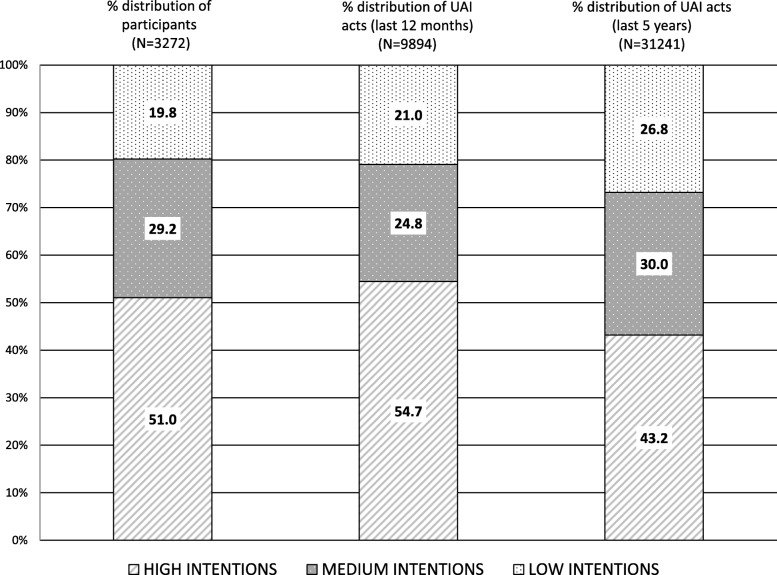
Distribution of participants and distribution of the total number of Unprotected anal intercourses (last 12 months and last 5 years) by testing intention

Of a total of 9894 at risk UAI acts occurred in the last 12 months, MSM with low intentions accounted for 21.0% of these acts. This contribution increased to 26.8% when we took into account the 31,241 of the at risk UA acts occurred in the last 5 years (Fig. 1).

### Reasons for never testing

Among individuals that had never been tested, risk reception related reasons (41.0%) were more frequently reported by those with low testing intentions (49.0%) than by those with medium (40.3%) and high testing intentions (34.3%). Not knowing where to test without being identified was reported more frequently by those with high (22.1%) and medium intentions (20.5%) than by those with low testing intentions (15.3%). Likewise, fear of consequences of a positive result was more frequently reported by those with high and medium intentions (11.7 and 11.2%) than by those with low intentions (6.9%) (Table 3).

**Table 3:** Main reason for never having been tested before by testing intention, in MSM at risk of acquiring or transmitting HIV

	High intentions		Medium intentions		Low intentions		Total		
	N	%	N	%	N	%	N	%	P* = 0.006
Risk perception related reasons**	141	34,3	212	40,3	192	49,0	545	41,0	
**Do not know where to go to get tested without been identified**	91	22,1	108	20,5	60	15,3	259	19,5	
Fear of consequences of a positive result***	48	11,7	59	11,2	27	6,9	134	10,1	
**Not wanting to go to a general practitioner**	39	9,5	52	9,9	31	7,9	122	9,2	
**Discomfort when answering intimate and personal questions**	34	8,3	43	8,2	41	10,5	118	8,9	
**Not wanting to wait several days to obtain the results**	17	4,1	11	2,1	7	1,8	35	2,6	
**Desire to test in a private center but lacking financial means**	6	1,5	13	2,5	3	,8	22	1,7	
**Others**	35	8,5	28	5,3	31	7,9	94	7,1	
**Total**	411	100,0	526	100,0	392	100,0	1329	100,0	

*p value: Chi-square test

**“I felt healthy” and “I thought that with my behaviours I could not be infected”

***It includes “fear of the consequences for my health”; “fear of losing my job or finding a new one”; “could have problems to obtain a work or residence permit”; “fear of rejection and discrimination”

## Discussion

In an online sample of MSM at risk of AUT HIV we identified a relevant group of MSM who reported having low intentions of seeking an HIV test in spite of having reported one of the highest UAI rates and the highest average time at risk of AUT HIV. Their low intention to actively seek an HIV test and low testing rates, suggest that this subpopulation of MSM is probably difficult to reach through CIT strategies.

Research on testing intentions among MSM is scarce in developed countries. To our knowledge, only two studies have been conducted to assess this aspect in Europe. In both studies [20, 21], nearly 4 in 10 MSM reported low testing intention doubling, the proportion observed in our study. In the study by Mackellar et al., part of the difference is explained by the fact that their analysis was restricted to never testers. In our study the percentage of never testers reporting low testing intentions is still lower but closer to the percentage presented by them (30% vs. 40%). In the one by Knussen et al., part of the difference could be explained by the fact that the study was conducted in 2000 when the expansion and normalisation of HIV testing had yet to occur.

In both studies [20, 21], higher numbers of UAI were associated with higher testing intentions. Conversely, in our analysis the number of UAI (last 12 months) reported by those with low testing intentions is as high as in those reporting high intentions. In our study population, testing intentions might not necessarily be mediated by risk exposure. This is a unique finding, since self-risk perception has been associated with testing intention in a number of studies [19]. However, risk behaviours are not always concordant with self-risk perceptions and some studies suggest that individuals can successfully identify risk for HIV in other individuals and/or groups but might not be as proficient when it comes to identifying their own behaviours as potentially risky [22, 23]. The fact that the most frequently reported reasons for not having been tested by at risk participants –especially in those with low testing intentions- were risk perception related is compatible with this idea.

By using a continuous variable to assess both risk per person (UAI per person) and time at risk (average time at risk of acquiring/transmitting HIV) we were able to assess the differences in risk behaviours per person in two different time frames. The differences of UAI per person in the last 12 months between the three testing intention groups is relatively small, however, because of more prolonged periods at risk, they are substantial when we consider the last 5 years. This approach provides relevant information to establish priority groups that could be contributing to the expansion of the epidemic. In this sense, even though the low intention group is the smallest in size (under 20%), they represent approximately 30% of the UA acts occurred in the last 5 years.

In our study, MSM with low testing intentions were less related to the gay scene than the rest of the participants, making them difficult to target by gay-oriented testing promotion campaigns. We raise the question of whether CIT strategies are the best way of testing this undertested- high-risk sub-group. In Spain, testing can be found free of cost in the health system and there is a network of sexual health clinics where people can test anonymously [24]. Testing is also conducted in community based organisations and in several in-pharmacy testing programs [25]. All these services rely on the initiative of the individual to actively seek an HIV test. For those with low testing intention, CIT strategies could be better suited. There are a number of initiatives of this nature such as routine testing in health areas of high or very high prevalence [26] or Indicator condition testing [27, 28] that should be taken into consideration to reach this population.

The results of the present study must be interpreted taking into account a number of limitations. We were unable to assess whether if the same person had participated more than once. However, the overall objective of the survey was clearly explained in the access screen and, given that no retribution was given in exchange for participation, it is highly unlikely that someone repeated the survey. Similarly, we cannot dismiss the possibility of bots responding to the survey. To maintain anonymity and confidentiality we did not collect IP addresses which would be the best way of detecting them. Again, this possibility is highly unlikely given that there was no retribution offered for responding.

The estimates “UAI per person in the last 5 years” and “UAI acts in the last 5 years” were calculated assuming that the number of UAIs were stable in time. We cannot rule out the possibility that the number of UAI can vary substantially from year to year but the only study we found that presents longitudinal data on the evolution of the indicator “number of UAI in the last 12 months”, supports our assumption since they found no statistically significant variation in the number of UAI in MSM at risk of acquiring HIV [29]. We were not able to ascertain if individuals were putting into practice risk reduction strategies such as serosorting and/or strategic positioning. Thus, those having more UAI might be doing so because they are only having anal intercourse with partners of presumably equal (negative) serostatus. However, the efficacy of serosorting and strategic positioning are limited [30–32]. The sample was recruited almost entirely from MSM accessing sexual networking websites. Online dating websites are a frequent way of socialising and meeting new sex partners among MSM but the generalisation of these results to the overall MSM population needs to be made with caution. In this sense, MSM identifying as gay and reporting more sexual risk behaviours could be overrepresented as has been previously reported [33]. Further studies using other recruitment methods are needed in order to gain knowledge about the validity of these results in MSM subpopulations who are not using gay dating websites/apps. Finally, we were not able to classify a large group of MSM by their risk of acquisition/transmission of HIV. Testing intention profile was similar to that of those who were at risk of AUT HIV but whether if their sexual behaviours actually placed them at risk merits further studies. The main characteristics of non-classifiable individuals vs. not at risk and at risk of AUT individuals are shown in Additional file 2.

## Conclusion

There is a substantial fraction of high risk MSM that could be contributing disproportionately to the expansion of the epidemic. In spite of reporting a high number of UAI and being very far from meeting testing recommendations, they reported low intentions of actively seeking testing.

## Supplementary information

**Additional file 1.** Survey questions used for the study in English.

**Additional file 2.** Main characteristics of respondents by risk of acquiring transmitting HIV.

## Data Availability

The datasets used and/or analysed during the current study are available from the corresponding author on reasonable request.

## Abbreviations

AUT: Acquired/unknowingly transmitted
CIT: Client-initiated HIV testing
ECDC: European Centre for Disease Prevention and Control
MSM: Men who have sex with men
UAI: Unprotected anal intercourse
