# Supplementary material for: High risk men who have sex with men in Spain are reporting low intentions of actively seeking HIV testing: results from a cross-sectional study
Source: BMC Public Health. 2020 Mar 27;20:398. doi: 10.1186/s12889-020-8440-3 (PMC7099777; doi:10.1186/s12889-020-8440-3)
Supplement: Supplementary file 1 — Additional file 1. Survey questions used for the study in English. [file 12889_2020_8440_MOESM1_ESM.docx]

**Survey questions used in this study**

**Age**

**¿How old are you?** ________

**Place of birth**

**Were you born in Spain?**

Yes

No, in a different country 🡪 which one?

**Educational level**

**What is the highest educational level you have achieved?**

None or primary education (education up to 12 years of age)

First level secondary or middle grade vocational training (the level that should be finished at 16 years of age)

Second level secondary or upper grade vocational training (the level that should be finished at 18 years of age)

University studies

Others: please specify

**Settlement Size**

**How many inhabitants does the place you have lived most of the last 12 months have (approximately)?**

More than 1.000.000

Between 500.000 and 1.000.000

Between 100.000 and 500.000

Between 50.000 and 100.000

Between 10.000 and 50.000

Under 10.000

**Sexual partners (lifetime)**

**The term “sexu” can apply to different concepts. Here, we consider ONLY those with vaginal, anal or oral penetration**

**According to this definition ¿With whom have you ever had sex?**

I have never had sex, neither with men or women

Only with women

More often with women but at least once with another man

Equally with men and women

More often with men but at least one time with a woman

Only with men

**Relation with gay culture**

**Which of the following situations best describes your relationship with the gay scene and culture.**

I am a member or collaborate with a gay association

I usually hang out in gay venues with my friends

I only go to gay venues to hook-up with someone

I’ve hardly been to gay venues.

**Sexual orientation**

**Currently, with which of the following options you feel more comfortable**

Heterosexual

Homosexual

Bisexual

Others: please specify

**Unprotected sex**

**With how many partenrs did you have unprotected anal intercourse? (In the last 12 months)**

None

One

Two

3-4

5-9

10-19

20-50

Over 50

**Testing history**

**How many times have you been tested for HIV?**

More than 20 times

15 to 20 times

10 to 15 times

6 to 9 times

3 to 5 times

2 times

1 time

Never

**Time since last test**

**When were you last tested?**

“XX” days/weeks/ months/years ago

**Testing intentions**

**How likely are you to seek an HIV test in the following 12 months?**

Very likely

Likely

Not sure

Unlikely

Highly unlikely

**Main reason for not testing for HIV**

**What is the MAIN reason for not having been tested before?**

I feel very healthy

I think that, with my behaviours, I cannot be infected

I am afraid of the consequences for my health.

I am afraid I might lose my job or not find one if the test is positive.

I do not have a residence or work permit, and I think that I might have problems to obtain one if the test is positive.

I have fear of rejection or discrimination.

I don't want to go to my family doctor/ health centre.

I don't know where to go to get tested anonymously or where no one can recognize me.

Having to wait several days to know the result stresses me out

I want to get tested in a private centre but I cannot afford it.

I feel discomfort answering intimate and personal questions.

Others [Specify].
