# Supplementary material for: High risk men who have sex with men in Spain are reporting low intentions of actively seeking HIV testing: results from a cross-sectional study
Source: BMC Public Health. 2020 Mar 27;20:398. doi: 10.1186/s12889-020-8440-3 (PMC7099777; doi:10.1186/s12889-020-8440-3)
Supplement: Supplementary file 2 — Additional file 2. Main characteristics of respondents by risk of acquiring transmitting HIV. [file 12889_2020_8440_MOESM2_ESM.docx]

| **Main characteristics of respondents by risk of acquiring transmitting HIV** | | | | | | |  |
| --- | --- | --- | --- | --- | --- | --- | --- |
|  | **Not at risk (N=944)** | | **Not classifiable (N=4125)** | | **At risk of acquiring/transmitting HIV (N=3311)** | |  |
|  | **N** | **%** | **N** | **%** | **N** | **%** | **p** |
| **Recruited at gay dating websites** | 881 | 93,3 | 3803 | 92,3 | 3077 | 93,0 | 0,378 |
|  |  |  |  |  |  |  |  |
| **Age (median)** | (33) | | (33) | | (32) | |  |
| <25 | 145 | 15,4 | 740 | 17,9 | 728 | 22,0 | <0,001 |
| 25-29 | 193 | 20,4 | 815 | 19,8 | 660 | 19,9 |  |
| 30-39 | 323 | 34,2 | 1324 | 32,1 | 1020 | 30,8 |  |
| 40-44 | 132 | 14,0 | 515 | 12,5 | 374 | 11,3 |  |
| >=45 | 151 | 16,0 | 731 | 17,7 | 529 | 16,0 |  |
|  |  |  |  |  |  |  |  |
| **Place of birth** |  |  |  |  |  |  | 0,285 |
| Spain | 808 | 85,6 | 3593 | 87,1 | 2891 | 87,3 |  |
| Latin-America | 75 | 7,9 | 302 | 7,3 | 259 | 7,8 |  |
| Other Country | 61 | 6,5 | 230 | 5,6 | 161 | 4,9 |  |
| **Study level** |  |  |  |  |  |  | <0,001 |
| <University | 320 | 34,0 | 1736 | 42,2 | 1693 | 51,3 |  |
| University | 621 | 66,0 | 2380 | 57,8 | 1605 | 48,7 |  |
|  |  |  |  |  |  |  |  |
| **Not related to gay scene** | 322 | 35,3 | 1653 | 41,3 | 1382 | 42,7 | <0,001 |
|  |  |  |  |  |  |  |  |
| **Self reported sexual orientation** |  |  |  |  |  |  | <0,001 |
| Homosexual | 805 | 85,5 | 3270 | 79,9 | 2778 | 84,1 |  |
| Hetero-bisexual | 137 | 14,5 | 822 | 20,1 | 525 | 15,9 |  |
|  |  |  |  |  |  |  |  |
| **Number of inhabitants of place of residence** |  |  |  |  |  |  | <0,001 |
| >1.000.000 | 351 | 37,5 | 1233 | 30,3 | 809 | 24,7 |  |
| 500.000-1.000.000 | 119 | 12,7 | 517 | 12,7 | 376 | 11,5 |  |
| >10.000-500.000 | 403 | 43,1 | 1925 | 47,3 | 1722 | 52,7 |  |
| <10.000 | 63 | 6,7 | 396 | 9,7 | 363 | 11,1 |  |
| **Past testing history** |  |  |  |  |  |  | <0,001 |
| < 1 year | 944 | 100,0 | 1739 | 42,2 | 0 | 0,0 |  |
| >=1 y <2 | 0 | 0,0 | 502 | 12,2 | 814 | 24,6 |  |
| 2-< 6 | 0 | 0,0 | 671 | 16,3 | 926 | 28,0 |  |
| 6 or more | 0 | 0,0 | 171 | 4,1 | 225 | 6,8 |  |
| Never tested | 0 | 0,0 | 1042 | 25,3 | 1345 | 40,6 |  |
| **Testing intentions** |  |  |  |  |  |  | <0,001 |
| High intentions | 770 | 81,7 | 2429 | 58,9 | 1681 | 50,8 |  |
| Medium | 107 | 11,3 | 979 | 23,8 | 969 | 29,3 |  |
| Low intentions | 66 | 7,0 | 714 | 17,3 | 657 | 19,9 |  |
